# Targeting XPO6 inhibits prostate cancer progression and enhances the suppressive efficacy of docetaxel

**DOI:** 10.1007/s12672-023-00700-8

**Published:** 2023-05-27

**Authors:** Huming Wang, Xiangyu Teng, Yuan Lin, Chao Jiang, Xin Chen, Ying Zhang

**Affiliations:** Department of Urology, The Second Affiliated Hospital of Anhui Medical University, No.678, Furong Road, Shushan District, Hefei, 230601 P.R. China

**Keywords:** Prostate cancer, Drug resistance, XPO6, YAP1, Hippo pathway

## Abstract

**Background:**

Although XPO6, one of the Exportin family members, functions in malignant progression of certain types of cancer, its role in prostate cancer (PCa) has not been elucidated. Herein, we investigated the oncogenic effect and clarified the downstream mechanism of XPO6 in PCa cells.

**Methods:**

We detected the expression level of XPO6 in PCa tissues by immunohistochemistry (IHC) and analyzed the correlation between clinicopathological characteristics and XPO6 level based on TCGA database. The effects of XPO6 in the proliferation and migration or resistance to docetaxel (DTX) in PCa cells were assessed using CCK8, colony formation, wound-healing and Transwell assays. Mice experiments were performed to investigate the role of XPO6 in tumor progression and DTX effect in vivo. Further, functional analysis of DEGs revealed the correlation of XPO6 with Hippo pathway and XPO6 could promote the expression and nuclear translocation of YAP1 protein. Furthermore, blocking Hippo pathway with YAP1 inhibitor leads to the loss of XPO6-mediated regulation of biological functions.

**Results:**

XPO6 was highly expressed and positively correlated with the clinicopathological characteristics of PCa. Functional experiments indicated that XPO6 could promote tumor development and DTX resistance in PCa. Mechanistically, we further confirmed that XPO6 could regulate Hippo pathway via mediating YAP1 protein expression and nuclear translocation thereby promoting PCa progression and chemotherapeutic resistance.

**Conclusion:**

In conclusion, our research reveals that XPO6 potentially function as an oncogene and promotes DTX resistance of PCa, suggesting that XPO6 could be both a potential prognostic marker as well as a therapeutic target to effectively overcome DTX resistance.

**Supplementary Information:**

The online version contains supplementary material available at 10.1007/s12672-023-00700-8.

## Introduction

At present, a main problem in the treatment of PCa is that, although the initial treatment is effective, most patients will inevitably progress to castration-resistance prostate cancer (CRPC). CRPC, especially metastatic CRPC is one of the most common malignancies of cancer-related death in men [1]. Additionally, CRPC is the main factor responsible for the failure of PCa therapy because of the natural or acquired drug resistance of CRPC, especially resistance to docetaxel, the gold standard therapy for metastatic prostate cancer [2]. The resistance to docetaxel represents one of the main factors responsible for the failure of prostate cancer therapy [2]. Therefore, it is of great of priority to clarify the underlying molecular mechanisms involving in docetaxel resistance and identify the promising therapeutic targets [3].

The nuclear transport proteins, including importins and exportins, may play a vital role in cancer through transporting key mediators of tumorigenesis across the nuclear membrane in cancer cells [4]. There have been many studies on the role of importins in cancers, but the research on exportins in tumors is still in the initial stage. XPO1 is the most widely studied nuclear exportin and mainly responsible for the nuclear-cytoplasmic transport of hundreds of proteins and various kinds of RNAs [5]. XPO1 is frequently overexpressed and /or mutated and acts as a carcinogenic drivers in human cancers, like lung cancer, colorectal cancer, lymphocytic leukemia, gastric cancer, gallbladder cancer, and prostate cancer [6–11]. Other nuclear exportins, such as XPOT (XPO3), XPO4 and XPO5, have been found to promote or suppress cancers, respectively [12–15]. XPO6 plays crucial roles in multiple biological functions, including transcriptional regulation [15], cell death and cell adhesion [16], memory formation [17], and cell differentiation [18]. Accumulating evidence implicates XPO6 in cancer progression. For example, reducing XPO6 levels in breast cancer cells drives anti-cancer effects via accumulating profilin-1 [15]. In addition, XPO6 could promote non-small-cell lung cancer progression by influencing necrotic cell death [16]. However, the role of XPO6 in PCa remain unclear.

The Hippo pathway is a regulator of cells and tissues development or differentiation that consists of a large network of proteins [19]. Yes-associated protein 1 (YAP1), a transcriptional co-activator, is a downstream target of the Hippo pathway [20]. When the Hippo pathway is on, inactive YAP1 phosphorylated by upstream regulators retained in the cytoplasm by binding to 14-3-3 and is subsequently degraded in a ubiquitin-proteasome-dependent manner [21]. By comparison, activated YAP1 translocates from cytoplasm to nucleus and interacts with the members of the TEAD family to induce target genes expression [22]. However, in the past few decades, relevance of the Hippo pathway with cancers has become an important study because of its significant control on cell growth and cell polarity [23].

In this study, we screened nuclear exportin members that significantly affect the proliferation of PCa cells. As a result, XPO6 was crucial for PCa cells growth in vitro and in vivo. Importantly, XPO6 was elevated in PCa tissues and was correlated with advanced pathological grades. Additionally, the manipulation of XPO6 expression could affect Hippo pathway and the sensitivity of PCa cells to DTX. In conclusion, our study suggests that XPO6 could be a potential target for PCa treatment.

## Materials and methods

### Bioinformatics analyses

 A total of 495 cases containing both gene expression data.

(HTSeq-Counts) and clinical information from the PRAD database were obtained from The Cancer Genome Atlas (TCGA) for further analysis. This study satisfied the publication requirements stated by TCGA (http://cancergenome.nih.gov/publications-/publicationguidelines).

### Cell cultures and treatment

PCa cell lines (DU145 and PC3) were purchased from American Type Culture Collection (ATCC, USA). Cells were routinely cultured in the RPMI-1640 supplemented with 1% penicillin/streptomycin and 10% FBS at 37 ºC under 5% CO_2_. DTX-resistant DU145 cell (DU145-DR) and DTX-resistant PC3 cell (PC3-DR) were kindly provided by Yu Lin (Shanghai Jiao Tong University School of Medicine, Shanghai, China). DTX was obtained from MedChemExpress (MCE, USA) and handled according to the manufacturer’s recommendations. The cells were treated with DTX for 24 h. After DTX treatment, follow-up assays were performed. Super-TDU (YAP1 inhibitor, #HY-P1727) was purchased from MedChemExpress (MCE, USA).

### Cells transfection and lentiviral constructs

Cells were transfected using Lipofectamine 2000 reagent (Invitrogen, USA) according to the manufacturer’s instructions. All siRNAs targeting XPO6 were designed and purchased from GenePharma Technology (Shanghai, China). The sequences of siRNAs used in this study were described in Table S1. Full-length XPO6 and XPO6-shRNA were cloned into PCDH-CMV-EF1A-T2A-PURO and PGMLV-hU6-MCS-CMV-ZsGreen1-PGK-Puro-WPRE vectors, respectively. Transient transfection of XPO6-expressing vector into cells was achieved using the Lipofectamine 2000 reagent according to the protocols. To generate stable knockdown cell lines, lentiviral particles equipped with shRNAs targeting XPO6 were purchased from Genomeditech (Shanghai, China). XPO6-shRNA oligonucleotide sequences are as follows: shNC: TTCTCCGAACGTGTCACGT; shXPO6: GUGCCUUUCACUGAGCAAATT. Stable cell lines were obtained after selection with 2 µg/ml puromycin (Beyotime, China) for 7 days. The efficiency of XPO6 knockdown and overexpression were evaluated by western blot and qRT-PCR.

### Proliferation assays

Proliferative abilities for PCa cells were assessed using CCK8 assay and colony formation assay. For CCK-8 assay, cells were seeded in 96-well plates at a density of 3 × 10^3^ cells per well and then administrated with CCK8 Kit (Dojindo, Japan) at indicated time points (0, 1, 2, and 3 days). The absorbance at 450 nm was measured. For colony formation assay, about 1500 transfected cells were seeded into 6-well plates and then cultured for 2 weeks. Then, cells were fixed with 4% paraformaldehyde and stained with 0.1% crystal violet for 15 min.

### Migration assays

Migration abilities for PCa cells was assessed using wound-healing assay and Transwell assay. For wound-healing assay, the transfected PCa cells were seeded in 6-well plates. When cell culture reached about 95% confluence, the cell monolayer was slowly scratched with a sterile 200 µl pipette tip. The wound was imaged at 0 and 24 h. For Transwell assay, 5 × 10^4^ PCa cells were seeded in serum-free medium in the upper chamber. The medium containing 20% FBS was added to the lower chamber. After cultured for 24 h in a 37 °C incubator, the cells were fixed in 4% paraformaldehyde and stained with 0.1% crystal violet. Cells in upper chamber were removed and the number of cells which traversed the membrane was determined under a microscope.

### Polymerase chain reaction (PCR)

A PrimeScript^™^ RT reagent Kit with gDNA Eraser (Takara, China) was used for genomic DNA erasure and reverse transcription. A TB Green^®^ Premix Ex Taq^™^ (Takara, China) was applied for the PCR assay following the manufacturer’s instructions. The target sequences of the primers are provided in Table S2. All the PCR experiments were performed on a QuantStudio 6 Flex system (Themo fisher scientific, USA).

### Western blot analysis

Cells were lysed with RIPA lysis buffer (Beyotime, China) and centrifuged at 15,000 g for 30 min. The supernatants were used for western blotting. Nuclear and cytoplasmic proteins were extracted using the Nuclear and Cytoplasmic Protein Extraction Kit (Beyotime, China) according to the manufacturer’s protocols. Protein extracts were separated by SDS-PAGE and probed with the corresponding antibodies showed in Table S3.

### Immunohistochemistry

The tissues were fixed in formalin and buried in wax. The tissue specimens were sectioned and placed on a slide for histochemical evaluation. Briefly, tissue was dewaxed, and heated with EDTA buffer for 30 min. The slides were incubated with appropriate primary antibodies in accordance with the standard antigen retrieval protocol.

### Immunofluorescence

Briefly, cells were fixed with 4% paraformaldehyde, permeabilized with 0.2% Triton X-100, and blocked with 5% BSA. Later, cells were incubated overnight with primary antibody, followed by fluorescent-labeled secondary antibody (Proteintech, USA). DAPI was then used for DNA staining. Image capture was performed by an inverted fluorescence microscope.

### Animal experiments

Sixteen BALB/c male nude mice (5 weeks old) were purchased from Shanghai Jihui Laboratory Animal Care(Shanghai, China)and housed in a specific pathogen-free environment for 1 week before use. The mice were randomly divided into two groups (n = 8/group). shNC or shXPO6 PC3 cells (1 × 10^6^ per mouse) were injected subcutaneously into the flanks of nude mice. Tumor volumes were measured every 7 days. Tumor volume (mm^3^) = length x (width)^2^/2. When tumors volume reached ~ 100 mm^3^, shNC or shXPO6 mice were randomly allocated into two groups (n = 4/group) and treated with or without DTX (5 mg/kg, once a week) via intraperitoneal injection for 28 days. Mice were then sacrificed, and the tumors were excised and weighed. Then tumor tissues were fixed with 10% paraformaldehyde for immunohistochemical analysis, and the tissues protein was extracted for western blot analysis. All experimental procedures followed the strict NIH guidelines and acquired ethical approval from The Second Affiliated Hospital of Anhui Medical University for Animal Research.

### Statistical analysis

All experiment were repeated at least three times in vitro, and all data were analyzed with the Graphpad Prism 8.0. These results are expressed as the mean ± S.D. Comparisons between two groups were performed using Student’s two-sided t-test. P < 0.05 was considered as statistically significant.

## Results

### Identification of XPO6 as a regulator of PCa cell proliferation

**Fig. 1:**
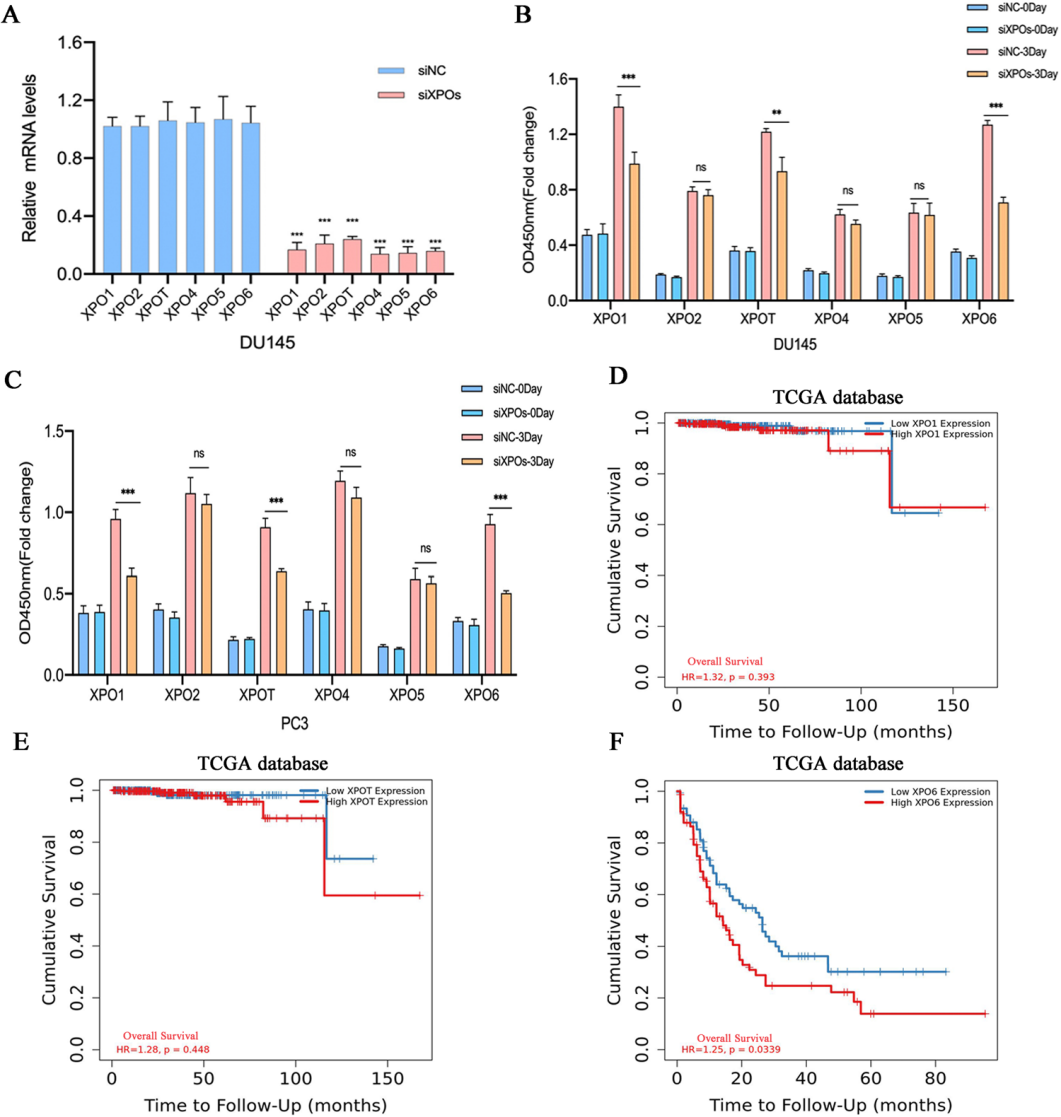
Screening of XPOs member as a regulator of PCa cell proliferation. **A** qRT-PCR was used to detect the silencing efficiencies of DU145 cell transfected with siRNAs targeting different XPOs members. **B**, **C** DU145 or PC3 cells viabilities were determined by CCK8 assay. **D**–**F** Cumulative survival analysis for XPO1, XPOT, and XPO6 in the Cancer Genome Atlas (TCGA) dataset. Data are mean ± SD (n = 3). *P < 0.05, **P < 0.01, ***P < 0.001

We initially screened six exportin family members whose functions in PCa development and progression have not been characterized but are predicted to be highly expressed in human PCa tissues. Each exportin member was silenced using siRNA oligonucleotides in PCa cells and the knockdown efficiencies were assessed by qRT-PCR (Fig. 1A). Subsequently, the cells viabilities of PCa cells transfected with siRNA were detected via CCK8 assays. The results demonstrated that XPO1, XPOT (also known as XPO3) and XPO6 silencing obviously decreased proliferation abilities of PCa cells (Fig. 1B–C). However, the survival analysis for these three genes suggested that only XPO6 was significantly correlated with the prognosis of PCa (Fig. 1D–F). Therefore, we chose XPO6 as our target gene for further study.

### XPO6 expression is upregulated and indicates a poor prognosis in PCa

**Fig. 2:**
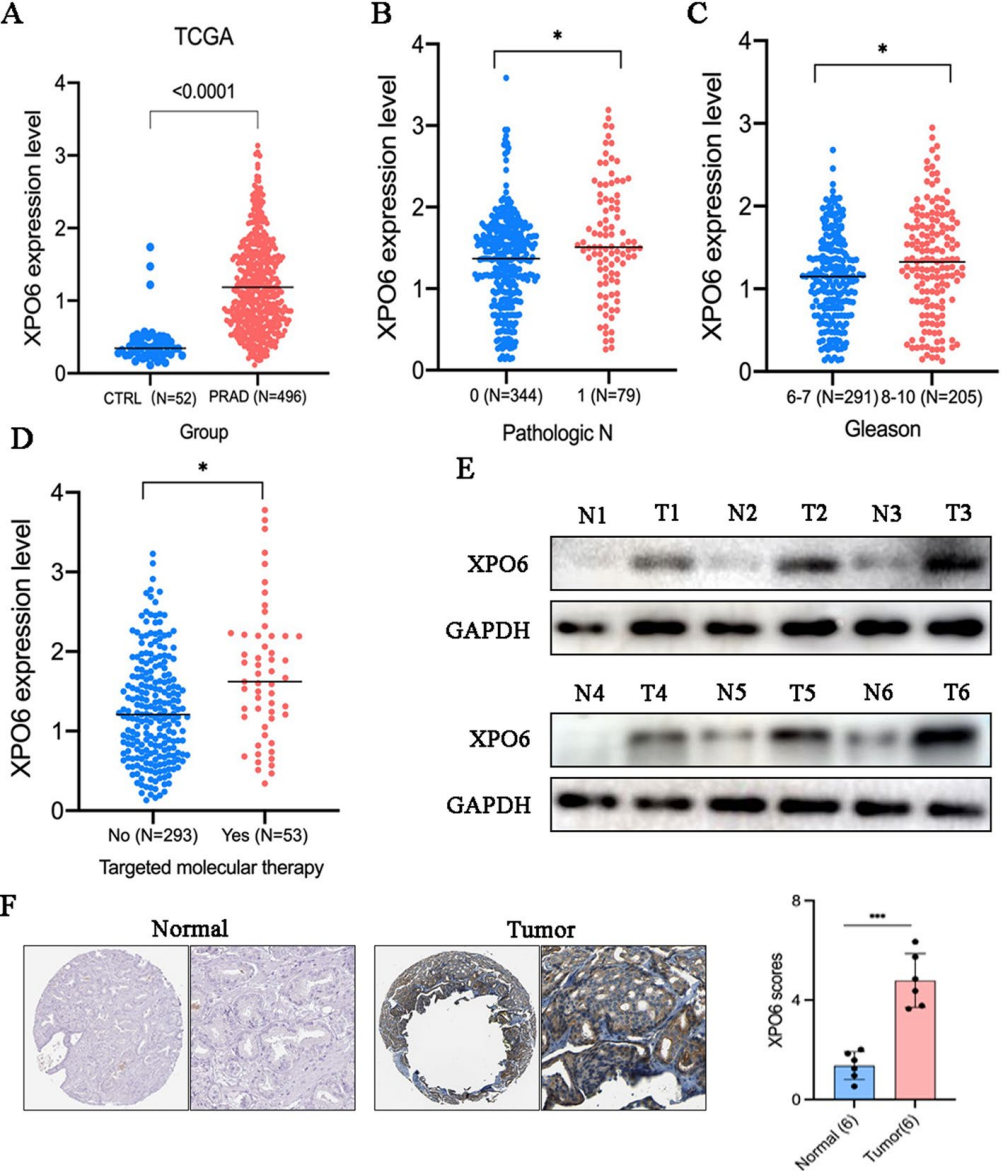
XPO6 expression analysis in PCa. **A** Comparison of XPO6 expression levels between normal prostate tissues and PCa tissues from TCGA dataset. **B**–**D** Comparison of XPO6 expression levels between N stages, Gleason scores, and targeted molecular therapy from TCGA datasets. **E** The immunoblotting for XPO6 expression of PCa tissues. **F** Expression of XPO6 in normal prostate tissues and PCa tissues was determined by IHC assay. Data are mean ± SD (n = 3). *P < 0.05, **P < 0.01, ***P < 0.001

We mined the TCGA database and found that XPO6 expression was up-regulated in PCa tissues compared to that in normal tissues (Fig. 2A). In addition, XPO6 was higher in advanced prostate cancer according to N classification, Gleason scores, and targeted molecular therapy or not (Fig. 2B–D). Logistic regression analysis demonstrated that higher XPO6 expression was correlated with poor prognostic characteristics (Table 1). High XPO6 expression in the PRAD cohort was significantly associated with N classification (OR = 1.71 for N1 vs. N0, P = 3.87E-02), Gleason score (OR = 1.76 for 8–10 vs. 6–7, P = 4.76E-02), targeted molecular therapy (OR = 2.54 for Yes vs. No, P = 2.54E-02), which was consistent with the correlation between XPO6 and clinicopathological characteristics of PCa (Table 2). Consistently, the protein levels of XPO6 were higher in PCa tissues than that in the normal prostatic tissues collected from 6 patients (Fig. 1E). Moreover, IHC results showed that the XPO6 expression was higher in tissues from PCa patients than normal tissues (Fig. 1F). These results reveal that XPO6 is upregulated in PCa and high XPO6 expression is more likely to be in an advanced stage than those with low XPO6 expression.

**Table 1:** XPO6 expression associated with clinicopathological characteristics (logistic regression)

Characteristics	Total (N)	Odds ratio (OR)	P value
Age	496	0.75 (0.52–1.06)	1.04E-01
Pathologic_M (M1 vs.M0)	457	0.51 (0.05–5.65)	5.82E-01
Pathologic_N (N1 vs.N0)	423	1.71 (1.04–2.82)	3.87E-02
Pathologic_T (T3&T4 vs.V2)	489	1.30 (0.93–1.83)	1.26E-01
Gleason score (8&9&10 vs.6&7)	496	1.76 (1.01–3.07)	4.76E-02
PSA ( = > 1 vs.<1)	439	1.19 (0.89–1.58)	2.45E-01
Targeted molecular therapy (Yes vs.No)	346	1.97 (1.09–3.58)	2.54E-02
Recurrence (Yes vs.No)	428	1.66 (1.25–2.91)	8.76E-02

**Table 2:** Correlation between XPO6 expression and clinicopathological characteristics of PCa

Characteristics total cases	N of case 496	XPO6 expression		P value
		Low (N = 248)	High (N = 248)	
Age(years)				
≤ 60	222	102 (20.56%)	120 (24.19%)	1.25E-01
> 60	274	146 (29.44%)	128 (25.81%)	
Pathologic_M				
M0	454	229 (50.11%)	225 (49.23%)	9.99E-01
M1	3	2 (0.44%)	1 (0.22%)	
Pathologic_N				
N0	344	176 (41.61%)	168 (39.72%)	4.54E-02
N1	79	30 (7.09%)	49 (11.58%)	
Pathologic_T				
T2	187	99 (20.25%)	88 (18.00%)	4.21E-01
T3	291	140 (28.63%)	151 (30.88%)	
T4	11	4 (0.82%)	7 (1.43%)	
Gleason score				
6–7	60	23 (5.15%)	37 (8.28%)	4.22E-02
8–10	387	202 (45.19%)	185 (41.39%)	
PSA value				
0–0.1	180	93 (21.18%)	87 (19.82%)	3.90E-01
0.1–1	212	106 (24.15%)	106 (24.15%)	
=>1	47	19 (4.33%)	28 (6.38%)	
Targeted molecular therapy				
Yes	53	19 (4.26%)	34 (7.62%)	2.79E-02
No	393	206 (46.19%)	187 (41.93%)	
Tumor recurrence				
Yes	59	23 (5.37%)	36 (8.41%)	9.21E-02
No	369	190 (44.39%)	179 (41.82%)	

### XPO6 promotes the proliferation and migration of PCa cells in vitro

**Fig. 3:**
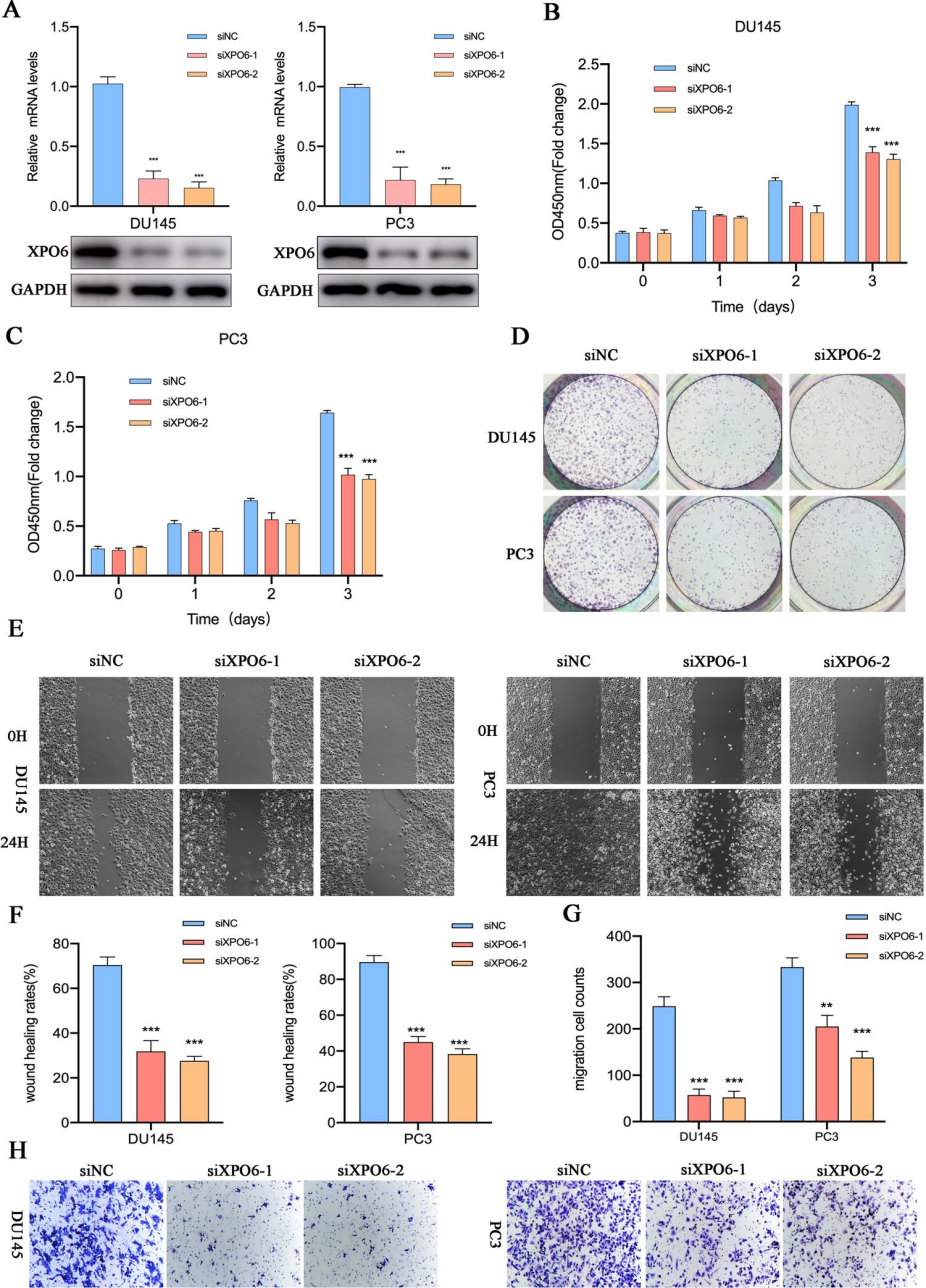
XPO6 inhibition suppresses the proliferation and migration of PCa cells. **A** qRT-PCR and western blot analysis showed the silencing efficiencies of XPO6 in DU145 or PC3 cells. **B**–**C** Cell viabilities was measured at the indicated time point in DU145 or PC3 cells. **D** Colony-formation assay showed the growth abilities of DU145 or PC3 cells after silencing XPO6 expression. **E**–**F** Wound-healing assay showed the migration abilities of DU145 or PC3 cells after XPO6 inhibition. **G–H** Transwell assay showed the migration abilities of DU145 or PC3 cells after XPO6 inhibition. Data are mean ± SD (n = 3). *P < 0.05, **P < 0.01, ***P < 0.001

To investigate the specific functions of XPO6 in PCa cells, we silenced XPO6 expression in DU145 and PC3 cells (Fig. 3A). CCK8 assays showed that the viabilities of both cells significantly decreased (Fig. 3B–C). Similar results were also obtained from the colony-formation assays (Fig. 3D). We next further evaluated the involvement of XPO6 in the migration behaviors of the PCa cells using wound-healing assay and Transwell migration assay. Consistent with the effects of XPO6 modulation on PCa cell proliferation, we found that knockdown of XPO6 significantly decreased the migration of both cells (Fig. 3E**–**H).

### Molecular signatures regulated by XPO6 in PCa

**Fig. 4:**
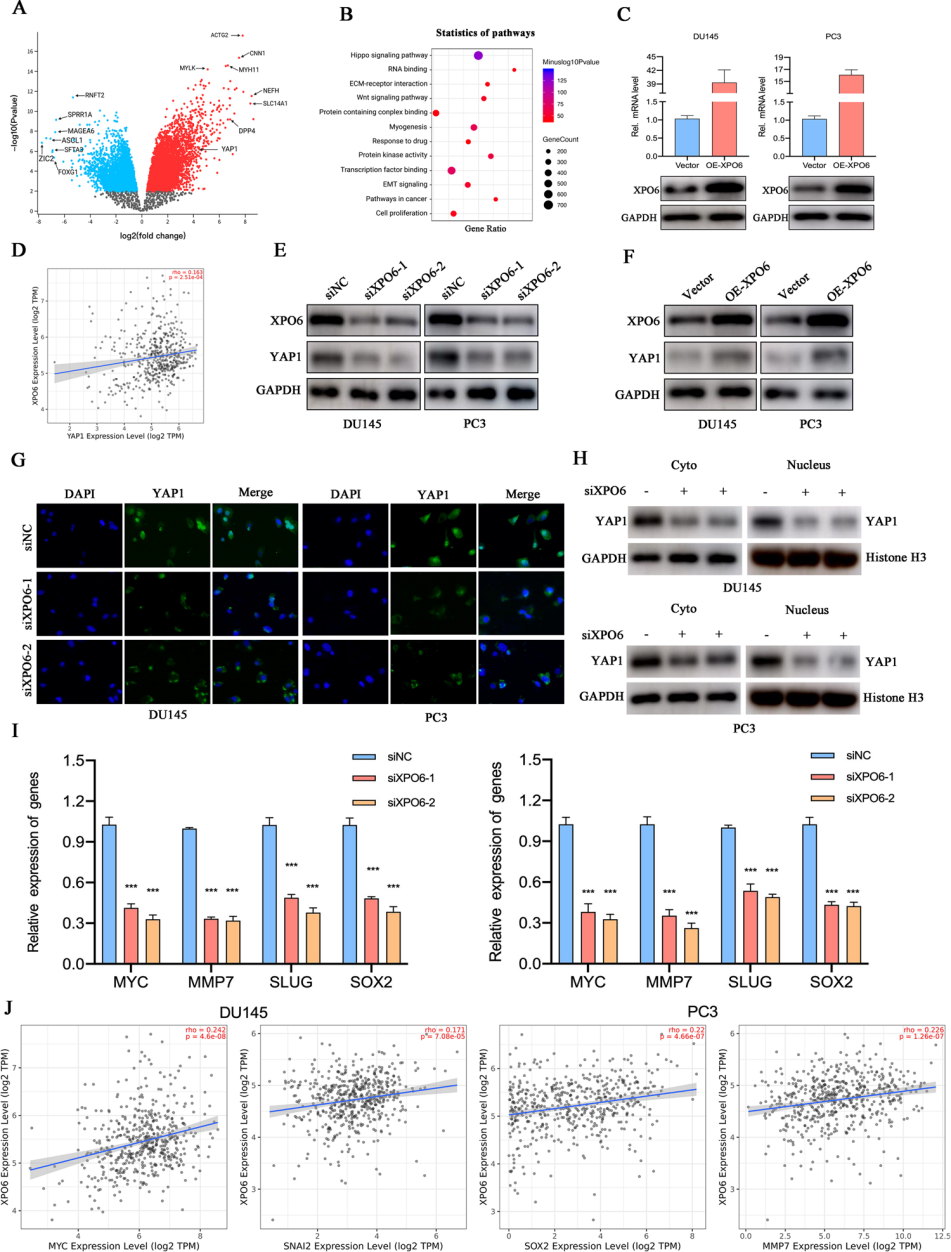
XPO6 was implicated with Hippo pathway and promotes YAP1 nuclear translocation. **A** The differentially expressed genes from TCGA PRAD dataset was presented as a volcano map. **B** The KEGG pathway annotation for differentially expressed genes was presented as a bubble diagram. **C** qRT-PCR and western blot were used to detect the overexpressing efficiencies of PCa cells transfected with XPO6-overexpression vector. **D** Correlation analysis between XPO6 and YAP1 was conducted based on TCGA database. **E** Western blot was used to analyze the expression of YAP1 in cells with XPO6 knockdown or overexpression. **F** Western blot was used to detect the expression of YAP1 in cytoplasm or nucleus in cells with XPO6 knockdown. **G** Immunofluorescence staining of YAP1 expression and location in PCa cells with XPO6 knockdown. **H** The mRNA expression levels of Hippo pathway targets MYC, MMP7, SLUG and SOX2 in cells were determined by qRT-PCR. **I** Correlation analysis between XPO6 and MYC, MMP7, SLUG and SOX2 was conducted based on TCGA database. Data are mean ± SD (n = 3). *P < 0.05, **P < 0.01, ***P < 0.001

To understand the molecular complexity, identify downstream targets, and the associated biological functions of XPO6 in PCa, we performed functional analysis of DEGs associated with XPO6 based on TCGA dataset. According to the cutoff values, samples in the TCGA dataset were divided into two groups (XPO6^high^ and XPO6^low^ groups). After comparison, over 941 differentially expressed genes (DEGs) were detected (*P* < 0.01, |log_2_FC| > 1.5), among which 584 genes were positively correlated with XPO6 and 357 genes negatively correlated, as shown in the volcano map (Fig. 4A). Then, The KEGG pathway enrichment analysis of the differential genes indicated that XPO6 is involved in the Hippo pathway, EMT pathway and cell proliferation pathway (Fig. 4B). The Hippo signaling pathway was significantly activated in this group.

### XPO6 promotes protein expression and nuclear translocation of YAP1

To validate above hypothesis, we first examined whether XPO6 regulates the expression of YAP1 by western blot analysis. We further constructed the XPO6-expression vector and the efficiencies of XPO6 overexpression were verified by western blot and qRT-PCR (Fig. 4C). Firstly, we conducted Correlation analysis based on TCGA database and found that YAP1 was positively correlated with XPO6 in PCa tissues samples (Fig. 4D). Indeed, YAP1 expression declined significantly with the knockdown of XPO6 in DU145 and PC3 cells, while its protein levels increased markedly with the overexpression of XPO6 in both cells (Fig. 4E–F). As is well known, the YAP1 exert its carcinogenic functions in cancers through the transcriptional activation of target genes in the nucleus. Therefore, we detected the level of YAP1 in the cytoplasm and nucleus using immunofluorescence. Indeed, knockdown of XPO6 in DU145 and PC3 cells led to relocalization of endogenous YAP1 from the nucleus to the cytoplasm (Fig. 4G). Consistently, Western blot analysis revealed that knockdown of XPO6 dramatically induced YAP1 in the cytoplasmic fraction, while it reduced YAP1 in the nuclear fraction (Fig. 4H). The expression of target genes of the Hippo pathway, such as MYC, MMP7, SOX2 and SLUG, decreased significantly in both DU145 and PC3 cells following XPO6 downregulation (Fig. 4I), which was consistent with the Correlation analysis results from TCGA database (Fig. 4J).

### Hippo signaling repression reversed enhanced phenotype induced by XPO6

**Fig. 5:**
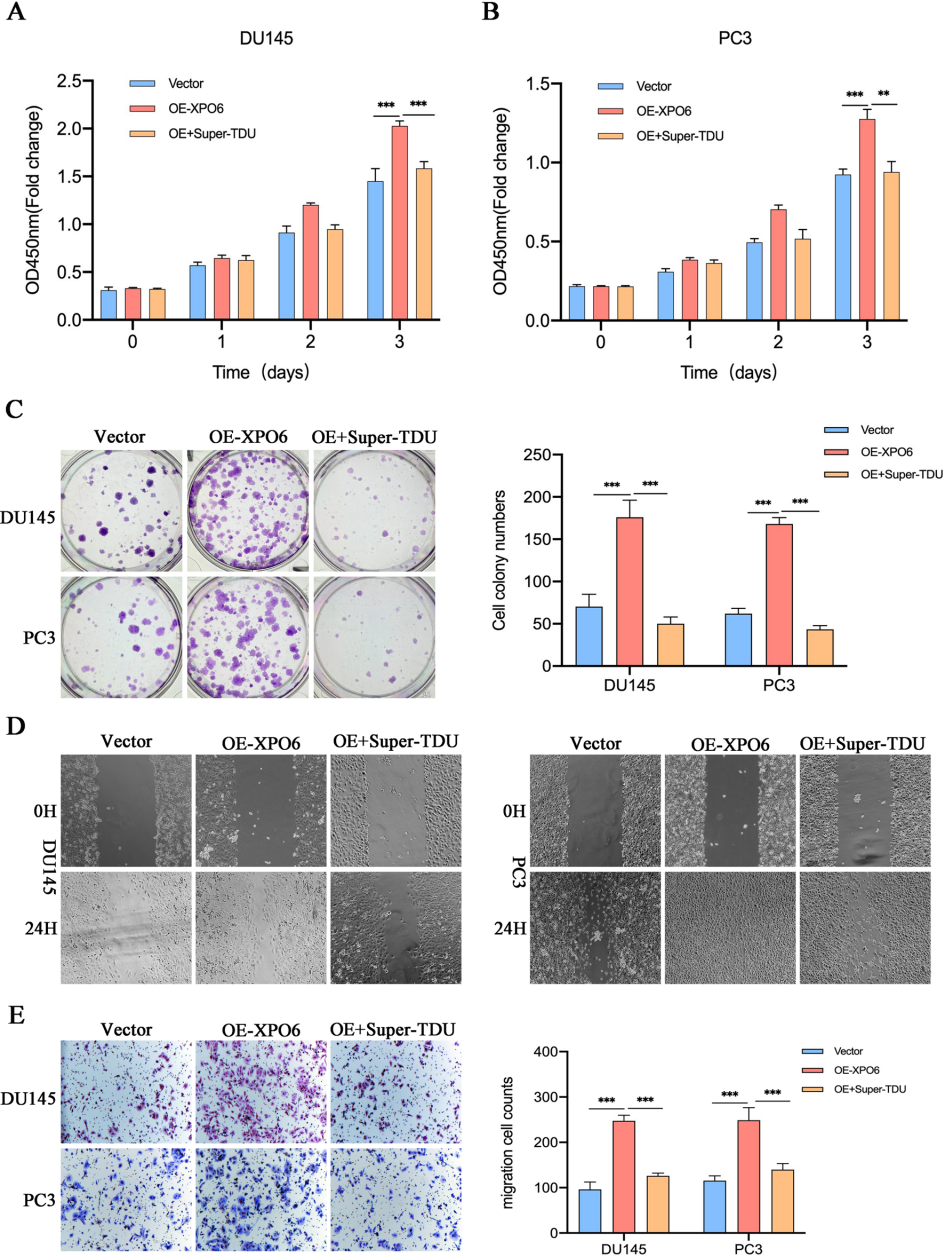
Molecular inhibitor of Hippo signaling reverses the enhancement of malignant phenotypes by XPO6. **A**–**C** CCK8 and colony formation assays were performed to detected cell proliferation capabilities of PCa cells treated with both XPO6 overexpression and Super-TDU. **D**–**E** Wound-healing and Transwell assays were performed to detected cell migration capabilities of PCa cells treated with both XPO6 overexpression and Super-TDU. Data are mean ± SD (n = 3). *P < 0.05, **P < 0.01, ***P < 0.001

The above experiments suggest that the suppression of XPO6 can regulate Hippo pathway by inhibiting the entry of YAP1 into the nucleus, which in turn inhibits the transcription of target genes the Hippo pathway. To further verify the reliability of this deduction, a series of rescue assays that used Super-TDU, a specific inhibitor of YAP1, were designed. DU145 and PC3 cells were divided into three groups based on whether they were transfected with the XPO6-expressing plasmid and were with or without the intervention of Super-TDU. Then, CCK8 and colony formation assays were performed. The results of these assays suggested that the addition of Super-TDU reversed the effects of XPO6 on proliferation abilities of the PCa cells (Fig. 5A–C). Similarly, Super-TDU could also reverse the effects of XPO6 on migration abilities of both cells (Fig. 5D–E).

### XPO6 inhibition reduced docetaxel resistance in the PCa cells

**Fig. 6:**
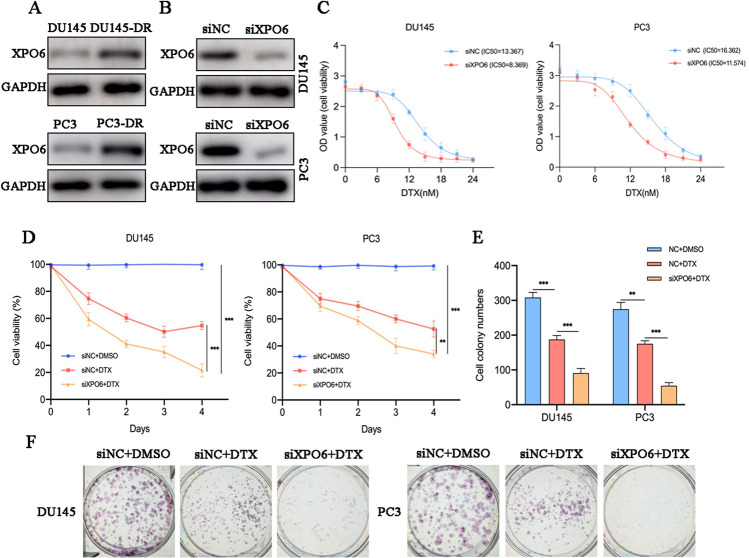
Downregulation of XPO6 inhibits the resistance of PCa cells to docetaxel. **A** Western blot was used to determine XPO6 expression in parental and DTX-resistance cells. **B** The knockdown efficiency of XPO6 was assessed via WB in PCa cells. **C** The cell viability of siNC and siXPO6 groups of the PCa cells under multiple concentrations of DTX intervention. **C** CCK8 assay was used to assess cell viability of PCa cells after DTX intervention (5µM) and/or siXPO6 treatment. **D** Colony-formation assay was used to detect proliferative ability of PCa cells after DTX intervention (5µM) and/or siXPO6 treatment. Data are mean ± SD (n = 3). *P < 0.05, **P < 0.01, ***P < 0.001

Accumulating evidence not only suggests oncogenic roles of Hippo pathway in cancer progression, but also its roles in chemotherapy resistance. For example, YAP1 could mediate DTX resistance in triple-negative breast cancer and its decreased expression could enhance the cytotoxicity of DTX and reverse drug resistance [24]. Similarly, YAP1 mediates DTX resistance in PCa [25–27]. On this basis, we estimated that XPO6 may also involve in DTX resistance in PCa because of its role in YAP1. Indeed, the protein expression of XPO6 was far more upregulated in the DU145-DR and PC3-DR cells than those in the parental DU145 and PC3 cells (Fig. 6A). First, we knock downed XPO6 levels in DU145 and PC3 cells (Fig. 6B). Then, the CCK8 assays were performed to assess the cell viabilities of XPO6 siRNA- and negative control siRNA-transfected DU145 and PC3 cells under various concentrations of DTX for 24 h. The IC50 values of the negative control siRNA-, XPO6 siRNA-transfected DU145 cells were 13.367nM and 8.369nM, respectively, whereas those of the negative control siRNA- and XPO6 siRNA-transfected PC3 cells were 16.362nM and 11.574nM, respectively (Fig. 6C). The IC50 values of the XPO6 siRNA-transfected DU145 and PC3 cells were markedly lower than that of negative control siRNA-transfected cells. In addition, the combination of XPO6 knockdown and DTX treatment revealed a stronger suppressive effect on cell proliferation in CCK8 assay (Fig. 6D). A colony formation assay was performed to validate these results. Under DTX treatment (5nM) there were less clones in the XPO6 knockdown group (Fig. 6E–F). Taking these findings together, it can be seen that the inhibition of XPO6 could significantly enhance the therapeutic effect of DTX.

### XPO6 suppression restrained PCa growth and enhanced the efficacy of docetaxel in mice

**Fig. 7:**
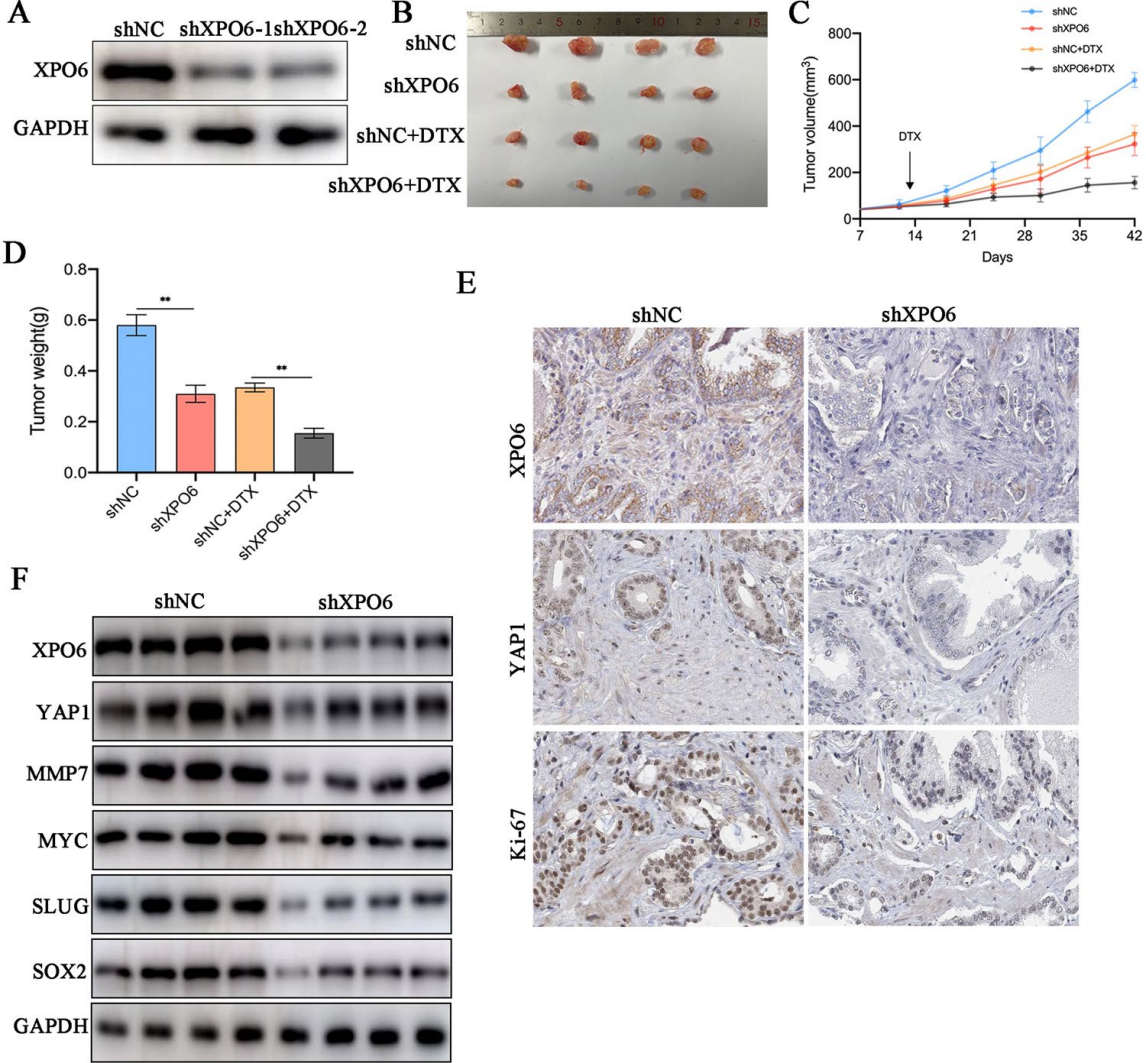
Knockdown of XPO6 suppresses the efficacy of docetaxel in vivo. **A** Western blot was used to determine the knockdown efficiency of shXPO7-transfected PC3 cells. **B** Representative images of the nude mice and xenograft tumors. **C** Curves of Tumor growth for XPO6-knockdown groups and controls with or without DTX treatment are analyzed. The tumor volumes were measured every 6 days. **D** Tumor weights of each group were measured after being surgically dissected. **E** Protein expression levels of XPO6,YAP1,MMP7,MYC,SLUG and SOX2 in nude mice were measured via western blotting. Data are mean ± SD (n = 3). *P < 0.05, **P < 0.01, ***P < 0.001

Experiments were performed to verify whether XPO6 knockdown could inhibit PCa tumor growth and enhance the efficacy of DTX in vivo. The nude mice were divided into four groups based on the presence or absence of XPO6 interference and the presence or absence of DTX treatment. The knockdown efficiency of XPO6 was assessed via western blot (Fig. 7A). It was observed that tumor size, volume, and weight were smaller in sh-XPO6 group or DTX treatment group than those in the control group, and XPO6 knockdown induced further reduction in the above parameters in group with DTX treatment (Fig. 7B–D). Subsequently, the expressions of XPO6, YAP1 and Ki-67 in shNC and shXPO6 groups of transplanted tumors were examined by performing immunohistochemical staining. The immunohistochemistry showed that the XPO6, YAP1 and Ki-67 expression levels of shXPO6-transduced PC3 xenografts significantly decreased than shNC-transduced xenografts (Fig. 7E). Consistently, Western blot analysis revealed that knockdown of XPO6 dramatically reduced XPO6, YAP1, MMP7, MYC, SLUG and SOX2 protein levels (Fig. 7F).

## Discussion

Docetaxel-based therapy is the first-line treatment for patients with progressive PCa. Though its great success in controlling the growth and metastasis of PCa, the emergency of chemoresistance limits its efficacy [28]. In eukaryotic cells, the nuclear membrane separates the cell contents into cytoplasm and nucleus, which ensures the presence of small molecules in their target subcellular compartments [29]. However, many transnuclear membrane proteins are dysregulated in cancers via various mechanisms, including altered expression of nucleocytoplasmic transporters [30]. XPO6 is the most recently discovered member of the exportin family and its upregulation is a prevalent cancer-associated event in PCa [31].

Here, we firstly described that XPO6 functions as a putative tumor-promoter in PCa. First, XPO6 expression is relatively higher in PCa tissues compared with the normal tissues. Second, higher XPO6 expression is correlated with several clinicopathological characteristics and poorer survival of PCa. Third, Functional experiments identified that targeting XPO6 suppresses PCa cells proliferation and migration in vitro and in vivo. More importantly, we identified XPO6 as a potential driver of ADT resistance. To clarify regulatory mechanism of XPO6, we performed functional analysis of DEGs associated with XPO6 in the TCGA database and found that the Hippo pathway was significantly correlated. Subsequently, we silenced XPO6 using siRNAs and confirmed the regulatory relationship between XPO6 and YAP1, MYC, MMP7 and other downstream target genes of the Hippo pathway using qPCR. XPO6 was found to regulate the Hippo pathway by promotes protein expression and nuclear translocation of YAP1, suggesting that YAP1 may be a substrate of XPO6. Furthermore, the suppression of Hippo pathway through YAP1 inhibitor treatment mitigated the regulatory effects of XPO6 on various phenotypes. Therefore, our results identified a new molecular mechanism conferring resistance to DTX and revealed potential therapeutic strategies against CRPC.

There are still certain limitations in this study. First, we found that XPO6 can regulate YAP1 protein levels, but we have little understanding of the mechanism by which XPO6 promotes YAP1 expression. The processes of transcription and translation involve a complex mechanism, which requires further exploration. Second, we found that XPO6 can promotes YAP1 nuclear translocation. However, it was, until now, unclear whether YAP1 was directed to the nucleus by direct interaction with XPO6. This will be an important question needs to address in future studies.

The Hippo pathway can be used as a target for cancer treatment. In addition, verteporfin has been reported to inhibit the YAP-TEAD complex formation and has been approved for use in animal research [32, 33]. Herein, we first reported that the dysregulation of the XPO6-YAP1 axis promotes PCa progression. Therefore, the development of inhibitor targeting XPO6 as agents for targeted therapy or combined treatment may offer new strategies for the treatment of PCa patients.

## Supplementary Information

Supplementary material 1

Supplementary material 2

Supplementary material 3

## Data Availability

The datasets generated and analysed during the current study are available from the corresponding author on reasonable request.

## Abbreviations

XPO6: Exportin 6
PCa: Prostate cancer
DTX: Docetaxel
YAP1: Yes-associated protein 1
DEGs: Differentially expressed genes
CRPC: Castration-resistant prostate cancer. PC3-DR:PC3 docetaxel-resistance
DU145-DR: DU145 docetaxel-resistance
