# Supplementary material for: Targeting XPO6 inhibits prostate cancer progression and enhances the suppressive efficacy of docetaxel
Source: Discov Oncol. 2023 May 27;14:82. doi: 10.1007/s12672-023-00700-8 (PMC10224898; doi:10.1007/s12672-023-00700-8)
Supplement: Supplementary file 1 — Supplementary material 1 [file 12672_2023_700_MOESM1_ESM.docx]

| **Table S1.** Sequences of siRNA oligonucleotides used in this study | | |
| --- | --- | --- |
| Gene | Sequence | |
| XPO1 | Sense | 5′- GCAGAUGCUUCCUUUAAAUTT-3′ |
|  | Antisense | 5′- AUUUAAGGAAGCAUCUGCTT-3′ |
| XPO2 | Sense | 5′- GUGCCUAACAUGGAAUUUATT-3′ |
|  | Antisense | 5′- UAAAUUCCAUGUUAGGCACTT-3′ |
| XPOT | Sense | 5′- CGGCCAAAUUCAAGAUACATT-3′ |
|  | Antisense | 5′- UGUAUCUUGAAUUUGGCCGTT-3′ |
| XPO4 | Sense | 5′- GGGCAAACUUAGUAAUUCATT-3′ |
|  | Antisense | 5′- UGAAUUACUAAGUUUGCCCTT-3′ |
| XPO5 | Sense | 5′- GAGCUGUAAUGGAGCAAAUTT-3′ |
|  | Antisense | 5′- AUUUGCUCCAUUACAGCUCTT-3′ |
| XPO6-1 | Sense | 5′- GGGAUUACAACAGUUUAUATT-3′ |
|  | Antisense | 5′- UAUAAACUGUUGUAAUCCCTT-3′ |
| XPO6-2 | Sense | 5′- GGGCUGAUCAUGUUGAAGATT-3′ |
|  | Antisense | 5′- UCUUCAACAUGAUCAGCCCTT-3′ |

**____________________________________________________________________**
