# Supplementary material for: Targeting XPO6 inhibits prostate cancer progression and enhances the suppressive efficacy of docetaxel
Source: Discov Oncol. 2023 May 27;14:82. doi: 10.1007/s12672-023-00700-8 (PMC10224898; doi:10.1007/s12672-023-00700-8)
Supplement: Supplementary file 2 — Supplementary material 2 [file 12672_2023_700_MOESM2_ESM.docx]

| **Table S2.** Sequences of primers used for qRT-PCR in this study | | |
| --- | --- | --- |
| Gene | Sequence | |
| XPO1 | Forward | 5′- AGCAAAGAATGGCTCAAGAAGT -3′ |
|  | Reverse | 5′- TATTCCTTCGCACTGGTTCCT -3′ |
| XPO2 | Forward | 5′- CAGAACACGCTGACAAGTATCT -3′ |
|  | Reverse | 5′- AGCCCTGCGTCTAGTATCAATA -3′ |
| XPOT | Forward | 5′- CAAGTCTTCGCCTTGCTTTTTG -3′ |
|  | Reverse | 5′- CCCTTGGATTTAGGTCCACTACT -3′ |
| XPO4 | Forward | 5′- CTGGAGAACGCGGCTAAAGTT -3′ |
|  | Reverse | 5′- CGCAGAGACTCGATGCTACC -3′ |
| XPO5 | Forward | 5′- ACACAGACAGAATTGGTGATGTT -3′ |
|  | Reverse | 5′- CAACTCCTACTCGACAGTTTGC -3′ |
| XPO6 | Forward | 5′- AACTCCTTTTGGCTCACCATAAA -3′ |
|  | Reverse | 5′- TGTCACAGGGGACTGGATCAA -3′ |
| MYC | Forward | 5′- GGCTCCTGGCAAAAGGTCA -3′ |
|  | Reverse | 5′- CTGCGTAGTTGTGCTGATGT -3′ |

| Gene | Sequence | |
| --- | --- | --- |
| MMP7 | Forward | 5′- GAGTGAGCTACAGTGGGAACA -3′ |
|  | Reverse | 5′- CTATGACGCGGGAGTTTAACAT -3′ |
| SOX2 | Forward | 5′- GCCGAGTGGAAACTTTTGTCG -3′ |
|  | Reverse | 5′- GGCAGCGTGTACTTATCCTTCT -3′ |
| SLUG | Forward | 5′- CGAACTGGACACACATACAGTG -3′ |
|  | Reverse | 5′- CTGAGGATCTCTGGTTGTGGT -3′ |
| GAPDH | Forward | 5′- CTGGGCTACACTGAGCACC -3′ |
|  | Reverse | 5′- AAGTGGTCGTTGAGGGCAATG -3′ |
