# Supplementary material for: Targeting XPO6 inhibits prostate cancer progression and enhances the suppressive efficacy of docetaxel
Source: Discov Oncol. 2023 May 27;14:82. doi: 10.1007/s12672-023-00700-8 (PMC10224898; doi:10.1007/s12672-023-00700-8)
Supplement: Supplementary file 3 — Supplementary material 3 [file 12672_2023_700_MOESM3_ESM.docx]

| **Table S3:** Antibodies used in this study | | |  |
| --- | --- | --- | --- |
| Target | Dilution | Company | Catalog Number |
| XPO6 (for WB) | 1:1000 | Abclonal | A10401 |
| YAP1 (for WB) | 1:1000 | Abclonal | A1002 |
| XPO6 (for IHC) | 1:500 | abcam | ab243712 |
| YAP1 (for IHC) | 1:100 | Abclonal | A1002 |
| Ki67 (for IHC) | 1:200 | abcam | ab15580 |
| MMP7(for WB) | 1:1000 | abcam | ab232737 |
| MYC(for WB) | 1:1000 | abcam | ab32072 |
| SLUG(for WB) | 1:1000 | abcam | ab27568 |
| SOX2(for WB) | 1:1000 | abcam | ab92494 |
| Histone H3 | 1:1000 | Bioworld | BS1174 |
| GAPDH | 1:5000 | abcam | ab8226 |
| Anti-Mouse Antibody | 1:2000 | CST | 7076 |
| Anti-Rabbit Antibody | 1:2000 | CST | 7074 |
